# Mortality of the elderly population during the COVID-19 pandemic. Are there north-south differences?

**DOI:** 10.25646/7061

**Published:** 2020-10-21

**Authors:** Enno Nowossadeck

**Affiliations:** Robert Koch Institute, Berlin, Department of Epidemiology and Health Monitoring

**Keywords:** EXCESS MORTALITY, CALENDAR WEEKS, COVID-19, NORTH GERMANY, SOUTH GERMANY

## Abstract

COVID-19 disease courses are dynamic and in some cases, fatal. In this article, we aim to identify the periods where overall mortality is higher, and therefore to more precisely measure excess mortality. We analysed mortality rate development for the population aged 65 years and older in Germany as a whole, a south Germany region (comprising the federal states of Baden-Wuerttemberg and Bavaria) and a north Germany region (comprising the federal states of Schleswig Holstein, Mecklenburg Western-Pomerania and Brandenburg). The article analyses the mortality rates per calendar week that have been published by Germany's Federal Statistical Office (Destatis) for the first 23 calendar weeks of 2020. We compare these figures with those for the same period 2016, the last year in which there was no influenza-related excess mortality. In calendar weeks ten to 15, mortality rates for the elder population rose exceptionally in the south compared to the north Germany region as well as compared to the 2016 figures. A peak was reached in calendar weeks 14 and 15. Mortality rates peaked around two to three weeks after incidence. Since this peak, mortality rates have decreased again, but up to calendar week 18 have remained above the 2016 rates. Overall the rise in mortality rates observed appears to be related to the COVID-19 pandemic and not the annual influenza wave.

## 1. Introduction

Since the beginning of 2020, the novel coronavirus SARS-CoV-2 (Severe Acute Respiratory Syndrome Coronavirus 2) has spread globally. Coronavirus disease 2019 (COVID-19), the disease caused by this virus, is a highly dynamic respiratory disease. Disease courses vary greatly, from asymptomatic to cases of severe pneumonia and death. This virus can, however, also affect further organ systems [1, 2] and thereby potentially causes fatalities that are not or not directly recognised as linked to COVID-19 developments. A full assessment of the pandemic, however, will require validated data on both the development of incidence (number of new cases) and mortality. A further option is to analyse so-called excess mortality, i.e. mortality that exceeds the expected figures.

COVID-19 cases are spread very unevenly across Germany. North German regions present far lower incidence rates than the south of the country. Presumably mortality is therefore also lower. This article analyses the development of general mortality in 2020 for Germany as a whole as well as for two regions, one in the north and one in the south of the country. The analysis concerns mortality in the population aged 65 years and older by calendar week. We limited ourselves to analysing mortality for the population aged 65 years and older because the vast majority of COVID-19-related deaths occurs in this age group. We compare these figures with 2016 mortality figures, the last year during which no influenza-related excess mortality was registered [3]. The (so far first?) wave of the pandemic peaked in Germany between February and April 2020, with the highest number of new cases being registered in March.

Analysing the extent of COVID-19-related and excess mortality requires defining the periods of increased general mortality. The objective of this article is therefore to identify the periods during which mortality in Germany as a whole as well as in the two analysed regions was higher in 2020 than in 2016.

## 2. Methodology

This article uses the weekly mortality figures published by Germany's Federal Statistical Office since 1 January 2016 for Germany and the individual federal states. In an attempt to at least partially compensate for random fluctuations, the north Germany region comprises the neighbouring states of Schleswig-Holstein, Mecklenburg Western-Pomerania and Brandenburg, with relatively low and similar incidence rates. The two states of Baden-Wuerttemberg and Bavaria constitute the south Germany region.

The analyses consider mortality for the age group 65 years and older. Mortality rates were calculated using the 1 January population figures for the corresponding age group for the years 2016 and 2020. Mortality rates were calculated as deaths per 100,000 inhabitants per calendar week on 1 January for the corresponding year. Current death figures were thereby reported to the Federal Statistical Office by each federal state in a special evaluation without conducting otherwise standard procedure plausibility checks [4]. This data should therefore be considered raw data. In addition, there was reporting delay. More reliable assessments for Germany as a whole are available with a delay of around four weeks, the point at which roughly 97% of the data for the most recently published calendar day is available. Yet this delay to providing the data varies greatly by region [4], meaning that for the purpose of this analysis, only data up to 7 June (end of calendar week 23), which had been published eight weeks later on 7 August 2020 by the Federal Statistical Office, was evaluated.

To identify the periods for analysis, a method to calculate joinpoint regression models was used. Based on this method, we can statistically define joinpoints where changes to trends over time occur. This allows not only to identify time points where a rising curve begins to fall (or inversely), but also the points at which the rate of a rising (or falling) tendency changes significantly. The joinpoint regression methodology also allows us to calculate average annual percent changes (APC) [5]. For consistency, all joinpoint calculations were based on a minimum of two joinpoints.

Average mortality rates per calendar week were then calculated for the identified time periods (total number of deaths over these periods per 100,000 inhabitants divided by number of calendar weeks). These average values were calculated to account for the different lengths of each of the identified time periods.

## 3. Results

### Germany

**Figure 1:**
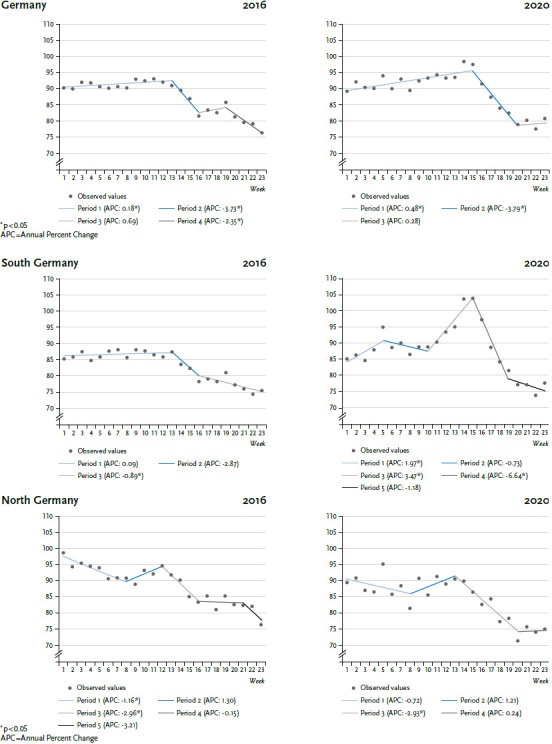
Development of mortality rates of the older population in Germany, the north Germany and the south Germany region 2016 and 2020 (joinpoint analysis results) Source: Statistical Federal Office (2020) [4], own calculations

Joinpoint analysis of mortality rates for the older population during the first 23 calendar weeks of 2020 (Figure 1) identified three periods: during the first 15 calendar weeks, mortality rates increased significantly (APC: 0.48%) and peaked in calendar weeks 14 and 15 (30 March to 12 April). After calendar week 16 (13 to 19 April), a strong decline is observed that continues until calendar week 20 (11 to 17 May). Mortality rates have stagnated since.

In 2016, mortality rates only increased until calendar week 13 (28 March to 3 April, APC: 0.18%). This was followed by a period of declining and/or stagnating mortality rates.

With an APC of-3.73% (2016) and -3.79% (2020), a more pronounced decrease of mortality rates is found during both years for the second half of the period analysed. In 2016, the joinpoint (the point at which the trend changes) was calendar week 13, whereas in 2020 it was later, in calendar week 15. Moreover, the decline of mortality rates began from a higher level (maximum 2020: 98.6 deaths per 100,000 inhabitants in calendar week 14) than in 2016 (maximum 2016: 93.2 deaths per 100,000 inhabitants in calendar week 11).

The per calendar day data on COVI D-19 deaths provided to the Robert Koch Institute (RKI) by public health departments show a rising tendency up to 8 April 2020, i.e. until calendar week 15. From there on, the number of COVID-19 deaths decreases until May [6].

For the population aged 65 years and older, mortality rates are not higher compared to 2016 and mortality also does not peak in calendar weeks 14 and 15.

### South Germany region

For 2020, a greatly modified pattern over time is evident comprising five distinct stages. During the first five calendar weeks, mortality rates increase significantly. This trend is then interrupted and, following calendar week ten (2 to 8 March), a strong and significant increase in mortality rates for the older population is observed (APC: 3.47%). These figures peak in calendar weeks 14 and 15 with 104 deaths per calendar week each per 100,000 inhabitants. This is followed by stage four characterised by a considerable decrease in the mortality rate (APC: -6.64%), a development which then flattens out in stage five.

In a direct comparison of the years 2016 and 2020, the decrease seen at the end of the observed calendar weeks in 2020 only began after calendar week 15, two weeks later than in 2016 (Figure 1). This decrease also began from a higher level.

### North Germany region

The situation in the north Germany region for the two years analysed is different (Figure 1): during the first period mortality rates drop up to calendar week eight (17 to 23 February), and then slowly (but not significantly) increase. During the third period, after calendar weeks 13 (in 2016) and 14 (in 2020), mortality rates initially go down considerably, whereby the differences in the magnitude of this drop are marginal (APC 2016: -2.96%; 2020: -2.93%).

No period during the second half of the period analysed in 2020 is found during which mortality rates in the north Germany region are considerably higher than at the beginning of the year. An observed slight increase following calendar week eight is not significant.

### Comparison between regions

Joinpoint analysis for south Germany 2020 indicate five distinct stages. Each of the first three stages takes five weeks (Figure 1), with the last two taking four. To compare figures between north and south, average calendar week mortality rates for each of these stages were calculated. The results are shown in Table 1.

During stage three, between calendar weeks eleven and 15, the mortality rate in south Germany was 97.4 per 100,000 inhabitants per calendar week for the age group 65 years and older. The 2016 mortality rate (85.2) was, by comparison, considerably lower. No differences between 2016 and 2020 for this period were registered for north Germany.

Also during the fourth period (calendar weeks 16 to 20), the average mortality rates per calendar week in south Germany were higher in 2020 than in 2016, even though they were already considerably lower than during the previous calendar week.

Most notably, during the three calendar weeks where mortality rates in south Germany were highest (calendar weeks 14 to 16), mortality rates in north Germany were lower than in south Germany (four to 17 deaths per 100,000 inhabitants), while mortality rates during the first calendar week 2020 had been higher in north Germany (two to five deaths per 100,000 inhabitants).

## 4. Discussion

This article analyses mortality for the population aged 65 years and older during the first months of 2020. The mortality rates analysed were for all deaths irrespective of the cause of death, i.e. not only those related to documented cases of COVID-19.

For the south Germany region, the analysis of mortality rates by calendar week for the older population reveals a considerably different pattern of rates during the first 23 calendar weeks of 2020 compared to 2016. Only in 2020 are very clearly rising mortality rates observed in calendar weeks ten to 15 with a peak in calendar weeks 14 and 15. This development is also seen, in albeit a weaker form, in the trend for Germany-wide figures for 2020. The characteristic pattern over time for the south Germany region for 2020 was not found in the north Germany region. This is therefore an extraordinary trend in the south Germany region, which was detected neither in the north Germany region nor in 2016 in either of the two regions. Consequently, we must assume an extraordinary situation for south Germany during the calendar weeks mentioned, which is presumably closely tied to the COVID-19 pandemic.

The following considerations would seem to support such an assessment:

First: incidence, i.e. the cumulative number of cases per 100,000 inhabitants is currently highest in the two federal states of Baden-Wuerttemberg and Bavaria (which together make up the south Germany region being analysed) [6]. From the outset of the pandemic, incidence in these federal states has been higher than in the northern region [7]. This means that the pandemic broke out earlier in these federal states than for example in the north Germany region, the second region analysed here. Higher mortality rates therefore occurred in the region where incidence was higher from the start of the pandemic.

This has also been linked to the region's proximity to Austria's ski resort of Ischgl: the further away a place was from Ischgl, the lower the incidence rate was. The town of Ischgl thereby stands for all north Italian and Austrian ski resorts in the Alps. The spread of the virus across Germany began in south Germany [8, 9].

Second: incidence peaked between 17 and 24 March 2020, i.e. in calendar week twelve. Calendar week twelve lies within the period which was identified with rising mortality rates (calendar weeks ten to 15). Assuming that the span between infection and death is around two to three weeks [10], the peak of incidence trends in the south Germany region fits with the subsequent peak of mortality rates. The COVID-19 mortality figures reported to the RKI also peaked in calendar week 15, which means that for south Germany in particular, the analysed trends for overall mortality rates follow a similar pattern to COVID-19 deaths.

The developments shown here for excess mortality are confirmed by developments in other European countries. An analysis by Vestergaard et al. found that excess mortality had peaked in calendar week 14. These evaluations also included data for Germany, however only from the states of Hesse and Berlin [11].

Non-pharmaceutical interventions (like maintaining social distancing and hygiene rules, wearing face masks and cancelling of large events, the closure of schools and childcare facilities and reducing contacts between people [24]) were introduced at the federal and federal state levels in March 2020 and led to a decline of incidence rates. After the assumption that non-pharmaceutical interventions can contribute to preventing or reducing COVID-19 mortality was made [12], the federal and federal state level non-pharmaceutical interventions in the north Germany region were introduced at an earlier stage of the pandemic resulting in an even lower number of infections. Possibly the north Germany region had an advantage because the pandemic reached this region later allowing the region to benefit from the experiences made in the south Germany region, leading a smaller number of people to become infected with COVID-19. Further factors that might have contributed to a milder course of the pandemic are the lower population density in combination with a less mobile, elderly population, the greater distance to ski resorts in the Alps (north Italy and Austria), as well the often lower socioeconomic status of people, which means that they do not go on two or more holidays per year or not as frequently (and therefore stay at home in winter). A further point to consider is the absence of super spreading events before lockdown implementation in the north Germany region. Super spreading events are events with a large number of participants to which a high number of infections with the virus can be traced. Such events have been documented for the rural districts of Heinsberg [13], Tirschenreuth [14], Rosenheim and Hohenlohekreis [15], all in the West and South of Germany. Super spreading events can play an important role in the spread of infectious diseases [16, 17]. For COVID-19 too, the international importance of super spreading events has been demonstrated [18–20].

Influenza may also have caused the increased mortality rates in the south Germany region in 2020 or be responsible for part of the rise, as is being discussed for the situation in the US [21]. Waves of flu have occurred repeatedly in Germany over recent years and have possibly prevented further increases to life expectancy [3, 22].

A fact contradicting the assumption that a wave of influenza is (at least in part) behind the increase in mortality rates found, is that the observed development occurred only following calendar week ten. The 2019/2020 flu wave, however, had already ended by calendar week twelve. Counting eleven weeks, this wave was shorter than the waves in the previous five seasons which took between 13 and 15 weeks [23]. Relative to previous seasons, respiratory infection rates also dropped abruptly after calendar week ten [24]. These results are taken from an analysis of GrippeWeb [24] data, a participatory internet-based syndromic monitoring tool used by the RKI to collect information directly from the population. More than merely reflecting changes in medical services use over the course of the COVID-19 pandemic, this data reveals actual epidemiological processes.

We interpret these developments as indicating the effectiveness of COVID-19-related social distancing measures (such as closure of child care facilities and schools, reduction of contact between people and maintaining distances) that aim to slow the spread of respiratory diseases [24]. As the flu wave subsided, mortality rates for the older population initially continued to rise, which means that we must assume that the increase in mortality rates seen in 2020 was not influenza-related.

It seems remarkable that, during the period where mortality rates were highest, mortality for the population aged 65 years and older in south Germany was higher than in the analysed north Germany region. Due to north-south differences in life expectancy [25], which need to be understood as expressing differences in social conditions [26, 27], we would have expected lower mortality rates in the south compared to the north Germany region. The phenomenon of a (temporary) ‘inversion’ of north-south differences in mortality points to the specific course of the spread of COVID-19 in Germany. During the early stages of the pandemic, the SARS-CoV-2 virus was brought to Germany mainly from ski resorts in the Alps. Regions with low levels of socioeconomic deprivation and people of relatively high socioeconomic status were hit harder because both travelling – in particular skiing trips – and participation in social events requires corresponding financial means [9]. This is a particular feature of the situation in Germany, as with other countries, the findings indicate that people in low socioeconomic status groups contract COVID-19 more frequently [28]. The extended course of the pandemic now makes it seem likely that in future in Germany, too, COVID-19 will hit people from low socioeconomic status groups harder [9]. Decreasing excess mortality in south Germany has led north-south differences to narrowing again.

Summing up the results of this analysis show that:

In March and April 2020, mortality rates for the population aged 65 years and older in Germany were temporarily increased; not so for the population aged under 65. In calendar weeks ten to 15, mortality rates for the older population in the south Germany region (federal states of Baden-Wuerttemberg and Bavaria) increased exceptionally in comparison to 2016 and also compared to the north Germany region (Schleswig Holstein, Mecklenburg Western-Pomerania and Brandenburg), the second region analysed. A peak was reached in calendar weeks 14 and 15. This constitutes a delay of two to three weeks relative to the peak of incidence, a fact explained by the average time between infection with COVID-19 and death. Since this peak, mortality rates have been declining again, but remained above the 2016 figures until calendar week 18 (27 April to 3 May). This means that the higher mortality rates observed in 2020 are most likely related to the COVID-19 pandemic and not the annual influenza wave.

## Key statements

- Mortality rates for the population 65 years and older in Germany were temporarily increased between March and April 2020.
- In calendar weeks 14 and 15 of 2020, considerably higher mortality rates for the older population were observed for the south Germany region.
- An increase in mortality rates was observed neither in the north Germany region in 2020 nor in south Germany in 2016.
- The mortality rate peaked in the south Germany region about two to three weeks after incidence.
- The increases seen in mortality rates were presumably related to the COVID-19 pandemic and not the annual influenza wave.

## Figures and Tables

**Table 1:** Average mortality rates per calendar week 2016 and 2020 for the population aged 65 years and older (deaths per 100,000 inhabitants on 1 January of each year) Source: Federal Statistical Office (2020) [4], own calculations

Calendar weeks	Germany		South Germany		North Germany	
	2016	2020	2016	2020	2016	2020
1–5	91.0	91.2	85.9	88.0	95.3	90.0
6–10	91.4	91.7	87.4	88.8	90.8	86.5
11–15	90.6	95.6	85.2	97.4	90.7	89.5
16–20	83.0	85.2	79.1	88.0	83.7	80.8
21–23	78.5	79.7	75.6	76.6	80.7	74.2
